# Nurses’ Role in Transitional Care During Intensive Care Unit Family Meetings for Patients With Prolonged Mechanical Ventilation

**DOI:** 10.1111/jan.70191

**Published:** 2025-09-01

**Authors:** HyunBin You, Sharron L. Docherty, Deepshikha C. Ashana, Wei Pan, Christopher E. Cox, Tolu O. Oyesanya

**Affiliations:** 1 School of Nursing, Vanderbilt University, Nashville, Tennessee, USA; 2 School of Nursing, Duke University, Durham, North Carolina, USA; 3 Department of Medicine, Duke University, Durham, North Carolina, USA; 4 Duke-Margolis Center for Health Policy, Duke University, Durham, North Carolina, USA; 5 Department of Population Health Sciences, Duke University, Durham, North Carolina, USA

**Keywords:** decision making, family meetings, intensive care units, nurse’s role, transitional care

## Abstract

**Aim:**

To describe nurses’ roles in transitional care planning during intensive care unit (ICU) family meetings for patients with prolonged mechanical ventilation (PMV).

**Design:**

A qualitative descriptive study.

**Methods:**

Using secondary data from a trial of a decision aid about PMV, transcripts from 19 unstructured ICU family meetings were purposively sampled and analysed using directed content analysis.

**Findings:**

Among 76 recorded ICU family meetings where nurses engaged and spoke at length beyond introduction, nurses spoke at length in 19 (25%) of them. These 19 family meetings were analysed in depth. Three themes were identified describing the roles nurses served: (1) Transitional care liaisons (e.g., introducing next levels of care, identifying/engaging family members, providing patient/family education, managing medications, planning for discharge, assessing patient/family needs, coordinating care, setting goals, providing care continuity, offering provider guidance and referring to resources); (2) information and communication facilitators (e.g., moderating family meetings, facilitating family understanding and serving as communication intermediaries) and (3) family support providers (e.g., providing emotional support, describing expectations and advocating for patients/families).

**Conclusion:**

Although nurses play a central role in patient care, they engage in only a minority of ICU family meetings addressing transitional care planning. Increased nursing involvement in these discussions may enhance care coordination and better support families navigating complex care transitions.

**Implications for Clinical Practice:**

Findings suggest that more consistent engagement of nurses in ICU family meetings has the potential to support transitional care planning and family-centred care for patients with PMV and their families.

**Impact:**

This work adds to a growing body of knowledge about nurses’ role in ICU transitional care planning. These findings provide valuable guidance for future research and development of transitional care standards to guide nurses in ICU transitional care planning.

**Reporting Method:**

The Consolidated Criteria for Reporting Qualitative Research Checklist (COREQ).

**Patient or Public Contribution:**

No patient or public contribution.

## 1 | Introduction

Patients experiencing prolonged mechanical ventilation (PMV), often defined as patients with chronic critical illness requiring mechanical ventilation for at least 10 days (Nelson et al. 2010), typically require intensive care unit (ICU) care and often lack decisional capacity (Cox and Carson 2012; Jubran et al. 2019). Families of patients with PMV often need to make high-stakes and value-laden decisions on behalf of the patient to meet the patient’s complex care needs (Dale et al. 2020). Many of these decisions occur during ICU family meetings; however, family members often feel unprepared to assist the patient during the ICU stay because of insufficient training, support and information (Dale et al. 2020; Davidson et al. 2012). Patients with PMV often have an extended ICU length of stay that complicates their recovery after hospital care; these patients also have multiple care transitions within the hospital and in post-acute care settings after the ICU stay (Damuth et al. 2015; Haines et al. 2021). These complexities underscore the importance of initiating transitional care in the ICU; however, the processes by which these early discussions occur warrant further investigation.

## 2 | Background

ICU family meetings occur between healthcare providers and families of the patients to share important information on the patient’s progress, treatment plans and care transitions. During these meetings, providers also engage the family members in shared decision-making processes (Azoulay et al. 2018; Davidson et al. 2016), which allows them to make healthcare decisions collaboratively, as well as share information on patient and family values, goals and preferences (Kon et al. 2016). Effective shared decision-making can improve psychological outcomes among families of ICU patients, such as decreased anxiety and stress and higher family satisfaction (Curtis and White 2008; Kon et al. 2016; White 2007). Although ICU clinicians regularly hold family meetings, topics related to transitional care (e.g., individualised discharge planning, goal setting, patient/family education, care coordination) are less frequently discussed compared to end-of-life care, palliative care and withdrawal of care (Garrouste-Orgeas et al. 2016). Additionally, family members take on complex care at home, coordinating care and advocating for their loved ones, often feeling unprepared for transitions in care (AARP and National Alliance for Caregiving 2020). ICU family meetings offer a valuable opportunity to initiate transitional care planning, enabling families to collaborate with healthcare providers and receive education and guidance (Nelms and Eggenberger 2010). Starting this process early, while patients are still in the ICU, could enhance coordination and delivery of care during and after the ICU stay (Hervé et al. 2020; King et al. 2019; Peters 2017). Despite the complex transitions expected for patients with PMV (Haines et al. 2021; Herridge and Azoulay 2023), there are no US ICU transitional care standards, and limited research has been conducted to discuss ICU transitional care planning in ICU family meetings.

Nurses play an important role on the ICU interdisciplinary team; nurses’ role in supporting ICU patients and their families is multifaceted (DeKeyser Ganz et al. 2016). Not only do nurses fulfil the information needs of the families of ICU patients but their relationship with the family members also provides emotional support by establishing trust and rapport (Adams et al. 2017; Davidson and Zisook 2017). Nurses also make significant contributions to family meetings. Nurses’ active participation in shared decision-making processes can facilitate improved provider–family communication, information sharing and care coordination and provide support for family members during patients’ ICU stay (Adams et al. 2017; Pecanac and King 2019; van Mol et al. 2017).

Prior research shows that nurses can effectively support and deliver transitional care planning to improve patient outcomes (Albert 2016; Bryant-Lukosius et al. 2015; Duncan et al. 2020; Mardani et al. 2020; Oyesanya et al. 2021). However, there are organisational barriers and a lack of resources for nurses supporting families of ICU patients in care transitions (McAndrew et al. 2020). Nurses report that their perspectives are often not fully heard or welcomed in multidisciplinary meetings (Wubben et al. 2021), including family meetings. While nurses’ role in the acute care of patients with PMV is clearly defined, nurses’ role in ICU family meetings, especially their role related to transitional care planning, remains less clear (Pecanac and King 2019). To address these gaps in knowledge, this study explored the role of nurses in transitional care planning in ICU family meetings for patients with PMV.

## 3 | Methods

### 3.1 | Design

We used a qualitative descriptive design to provide a holistic picture of the role of nurses related to transitional care in ICU family meetings. This study adhered to the Consolidated Criteria for Reporting Qualitative Research (COREQ) reporting guidelines (Supporting Information S1) (Tong et al. 2007).

### 3.2 | Data Source

This study was a secondary analysis of data from a randomised clinical trial to support decision-making for patients with PMV (Cox et al. 2019). The trial was conducted in 13 medical and surgical ICUs at 5 academic and community hospitals in North Carolina, Pennsylvania and Washington between 2012 and 2017. Study procedures of the parent study were published elsewhere previously (Cox et al. 2019).

### 3.3 | Inclusion and Exclusion Criteria

The eligibility criteria for patients were: (1) ≥ 18 years, (2) no anticipation of death or weaning from mechanical ventilation within 24 h and (3) ventilation for at least 10 days, which is often when placement of tracheotomy and the surgical feeding tube is considered (Nelson et al. 2010). Patients were excluded if they had: (1) decisional capacity, (2) no identifiable family caregiver, (3) clear preference for comfort care, (4) ventilation for more than 21 days, (5) chronic neuromuscular disease or (6) plans for organ transplantation. Eligibility criteria for family members included: (1) associated with a patient who met all aforementioned patient criteria, (2) ≥ 18 years of age and (3) self-identified as the most involved participant in the patient’s decision-making. Family members were excluded if they had difficulties listening and speaking in English and became ineligible if patients regained decisional capacity, were extubated or died during the study period. Each patient’s ICU physician (attending or fellow) and nurse who was present on the day of each patient’s family meeting were enrolled in the parent study.

### 3.4 | Sampling

We purposively sampled the transcripts of unstructured ICU family meetings where nurses were involved. The inclusion criteria for family meeting transcripts were (1) ≥ 1 nurse present at the meeting, (2) discussed topics related to transitional care (e.g., discharge planning, transitions of care, post-discharge care settings, post-discharge care needs, post-discharge quality of life and health outcomes) and (3) nurses spoke beyond introducing themselves. ICU family meeting transcripts were excluded if the family meeting transcripts (1) were incomplete, (2) were not conducted in English or (3) did not have a nurse speaking.

**FIGURE 1 |:**
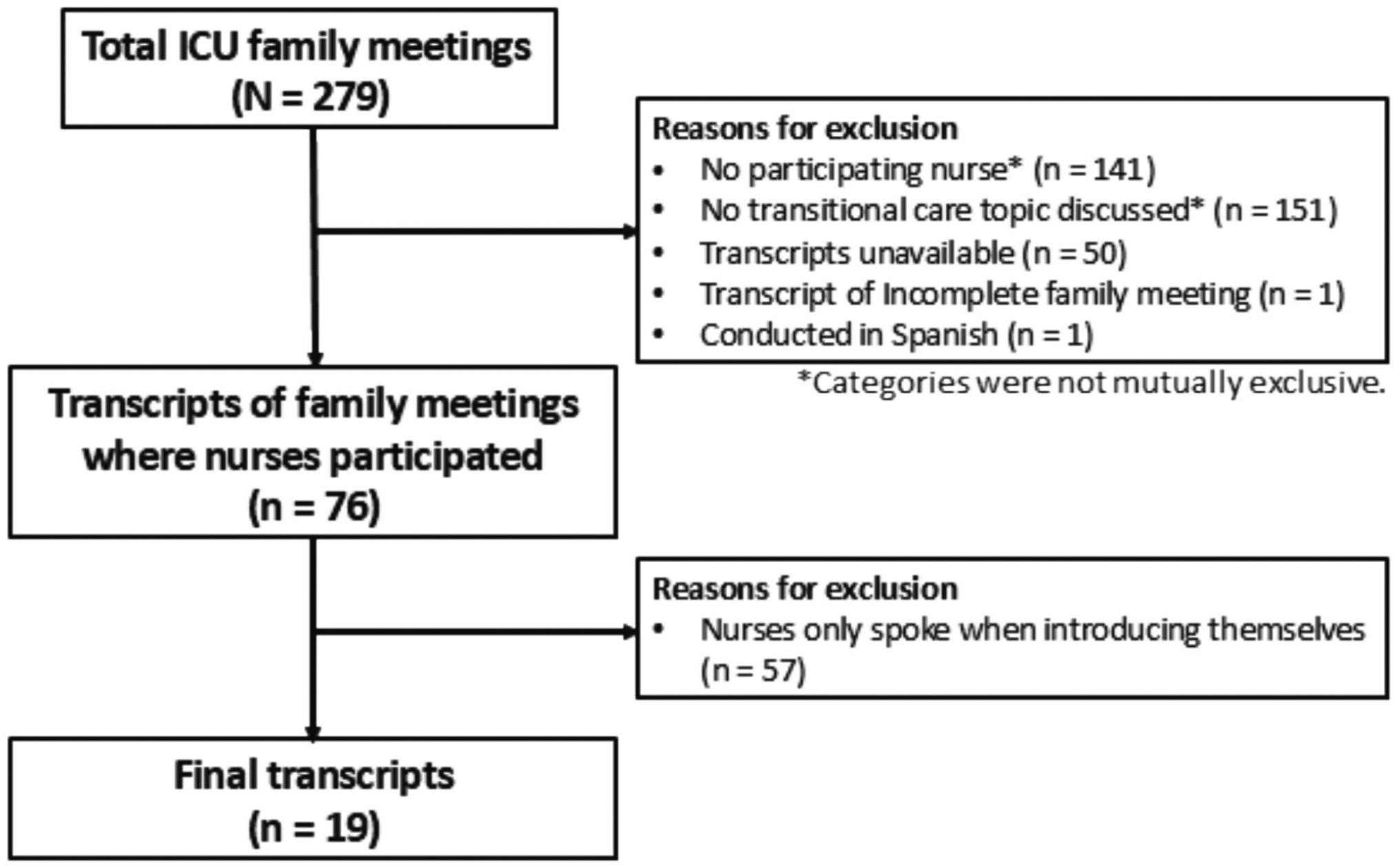
Purposive sampling flow diagram.

From a total of 279 ICU family meetings, there were 138 (49.5%) meetings where at least one nurse was present. A total of 128 (45.9%) family meetings discussed transitional care–related topics. Of these 128 meetings, 76 were conducted and audio-recorded in English. Of these 76 meetings, nurses spoke at length beyond introducing themselves in 19 (25%) meetings, thus leading to a final sample of 19 ICU family meetings (Figure 1).

### 3.5 | Data Collection

Sociodemographic characteristics of the participants, including patients, their primary family members and nurses, were collected at baseline. Healthcare providers, including physicians (e.g., attendings, fellows), nurses (e.g., bedside nurses, nurse coordinators) and social workers, were present at the family meetings to discuss topics related to the patient’s prognosis, treatment plans, goals, family members’ understanding of the care plan, etc. At least one family member per patient attended. The ICU family meetings were audio-recorded and transcribed verbatim. After each meeting, research staff collected meeting-related information, such as meeting participants and the topics discussed.

### 3.6 | Data Analysis

Descriptive statistics were used to tabulate ICU family meeting characteristics (e.g., nurse/family presence at meetings, meeting length, number of instances/words nurses spoke during meeting) and participant characteristics.

We used directed content analysis, which allowed the researchers to analyse the data using predetermined codes (Hsieh and Shannon 2005) to explore the content of the ICU family meeting transcripts specifically, focusing on nurses’ roles and nurse engagement related to transitional care. Directed content analysis has three phases: preparation, organising, and reporting (Hsieh and Shannon 2005). The data preparation phase began with selecting the unit of analysis, which was the response from nurses in each family meeting.

In the organising phase, predetermined codes were developed by adapting findings from the literature on nurse–family communication during the ICU family meeting, which include the strategies and characteristics of nurse engagement in ICU family meetings (Adams et al. 2017; Pecanac and King 2019). Our predetermined codes initially focused on (1) the manner in which nurses enter into the family meeting discussion (e.g., interruption, collaborative completion, responding to questions, asking a question) (Pecanac and King 2019), (2) the role of ICU nurses (Adams et al. 2017; Davidson 2010) and (3) topics related to transitional care planning discussed by nurses (Chaboyer et al. 2005; Hervé et al. 2020). Newly emerging codes were added if they did not fall into predetermined codes (Hsieh and Shannon 2005). Latent content was also captured to fully discover the ‘underlying’ meaning, context and nuances of the participants’ responses (Hsieh and Shannon 2005). The first and senior authors coded the data, using Microsoft Word, with the first author coding 100% of the data independently and the senior author auditing 60% of the first author’s coding. Coding disagreements were discussed until consensus was met. A codebook, including code names, definitions and quotation exemplars, was developed and revised iteratively throughout the analysis. Finally, in the reporting phase, the findings of this study were displayed using thick description with quotation exemplars.

### 3.7 | Ethical Considerations

Approval for the study was granted on 10 January 2010, by the Duke University Health System Institutional Review Board (IRB# Pro00021965) before the study commencement. All participants signed a written informed consent. All family meeting data were coded to ensure the confidentiality of the participants.

### 3.8 | Rigour and Reflexivity

Several strategies were utilised to ensure the rigour and trustworthiness of the findings. Credibility, defined as addressing the congruence of the findings with reality, was achieved through peer examination with research team members (Tracy 2010). Dependability, known as ensuring that results can be repeated or that findings remain stable over time, was obtained by having an open dialogue between the team members throughout the process to reach a consensus in coding transcripts (Shenton 2004). Confirmability, which ensures each stage of data collection, analysis decisions and coding processes were not influenced by the investigator’s bias, was increased by using quotation exemplars as evidence of our findings (Shenton 2004). Transferability, which allows applications to other contexts, settings or groups, was addressed by providing detailed descriptions of the sample, research procedures and analysis (Shenton 2004). Furthermore, an audit trail and reflexive journaling were used throughout the study to document the thought processes and decisions made during the analysis stage to avoid bias from using a directed approach.

### 3.9 | Team Member Background

Our research team consisted of three female PhD-prepared nurses (first, second and senior author); of those, two had a clinical background in critical care (first and second author), one female critical care physician (third author), one male critical care physician (fifth author) and one male PhD-level biostatistician (fourth author), with expertise in critical care or qualitative research. First, second, third and senior authors had expertise in qualitative research. The first author read family meeting transcripts multiple times for accuracy and to become familiar with the data and obtain a sense of the context and topics.

## 4 | Findings

### 4.1 | Characteristics of Participants

#### 4.1.1 | Family Meeting Characteristics

Family meeting characteristics are described in Table 1. The 19 family meetings were held for a median length of 47 min (IQR = 35–55; range 23–69), with a median of one (IQR = 1–1) nurse and three (IQR = 3–5) family members participating in each family meeting. Across all family meetings, there were a total of 23 nurses (not unique), who identified themselves as nurse coordinators (*n* = 12, 52.2%), bedside ICU nurses (*n* = 3, 13%) or unspecified (*n* = 8, 34.8%). Each of the nurses in this sample participated in a median of one (IQR = 1–2) family meeting, ranging from 1 to 6. On average, nurses spoke a median of 13 times (IQR = 9–22) and 561 words (IQR = 250–929) per family meeting. The most frequently discussed transitional care–related topics were discharge dispositions (*n* = 19, 100%), discharge needs (*n* = 17, 89.5%), post-discharge quality of life (*n* = 17, 89.5%) and functional status (*n* = 16, 84.2%). Of the 19 family meetings, 68.4% of the meetings were held at one institution that had a nurse coordinator role, whereas the other three study sites did not have this nursing role.

#### 4.1.2 | Meeting Participant Characteristics

This study’s sample comprised 19 patients with PMV and their primary family members and 12 unique nurses who participated in the 19 family meetings. Table 2 describes the characteristics of nurses and families of patients with PMV.

Patients were mostly White (*n* = 10, 52.6%) males (*n* = 10, 52.6%), admitted to medical ICU (*n* = 15, 78.9%), with a mean age of 55.5 years (SD = 12.84), an APACHE II illness severity score of 25.2 (SD = 5.85) and a Charlson comorbidity score of 3.9 (SD = 2.68). Similarly, family members were mostly White (*n* = 10, 55.6%) females (*n* = 9, 50.0%), with a mean age of 51.1 years (SD = 12.26), identifying as the patient’s spouses or partners (*n* = 8, 44.4%), children (*n* = 4, 22.2%) or other families (*n* = 3, 16.7%) of patients. The nurses who participated were all White (*n* = 8, 100%; *n* = 4 did not report) females (*n* = 10, 100%, *n* = 2 did not report), with a mean age of 43.5 years (SD = 10.81; *n* = 4 did not report).

### 4.2 | Themes

**FIGURE 2 |:**
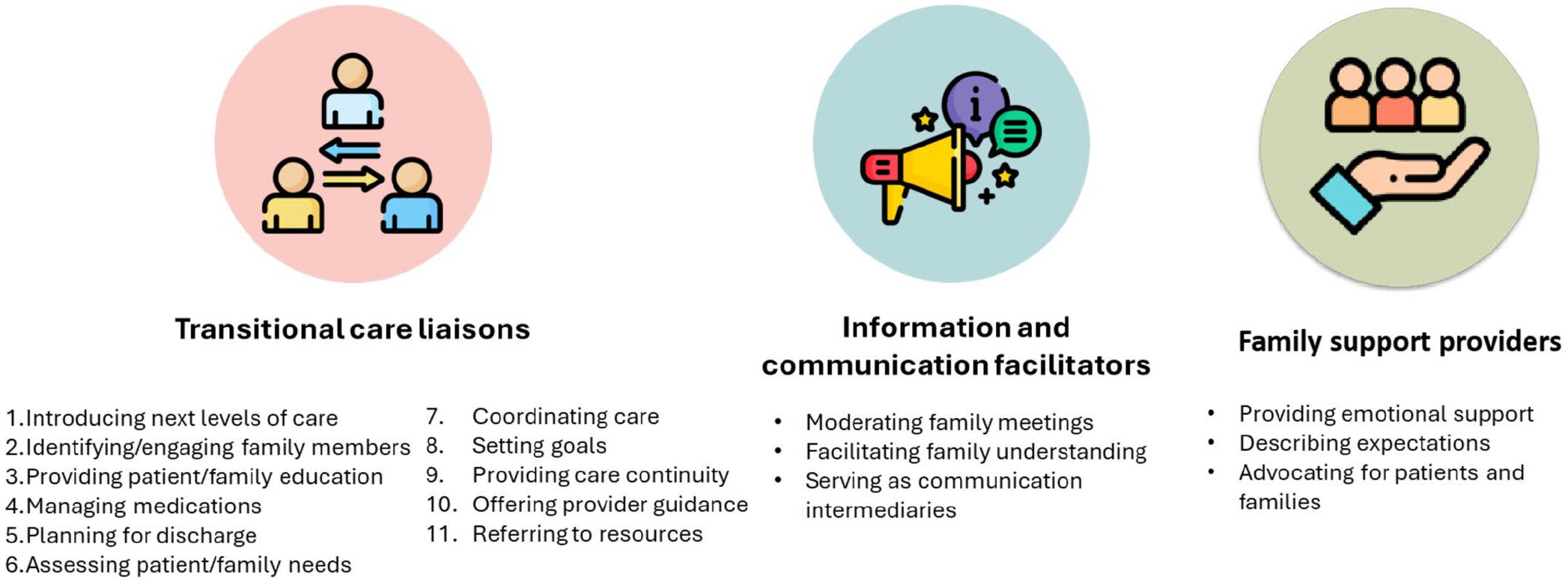
Nurses’ role in transitional care planning in ICU family meetings.

The 19 ICU family meetings occurred with a range of family members and interdisciplinary healthcare providers present, including physicians, nurses, pharmacists, social workers, case managers and chaplains. Though multiple people spoke in these meetings, per the aim of the study, we focused on what nurses shared and how they engaged in these meetings. Nurses either volunteered information without prompting or someone (another healthcare provider or a family member) invited the nurse as the next speaker, often by asking a question. Three themes were identified: (1) nurses as transitional care liaisons, (2) nurses as information and communication facilitators and (3) nurses as family support providers (Figure 2).

#### 4.2.1 | Theme 1: Nurses as Transitional Care Liaisons

In family meetings, nurses engaged in multiple strategies to coordinate seamless patient care transitions in anticipation of transfer from one level of care to another, ultimately serving as transitional care liaisons. Nurses used 11 transitional care strategies while engaging in transitional care planning (in no specific order), including (1) introducing the next levels of care, (2) identifying/engaging family members, (3) providing patient/family education, (4) managing medications, (5) planning for discharge, (6) assessing patient/family needs, (7) coordinating care, (8) setting goals, (9) providing care continuity, (10) offering provider guidance and (11) referring to resources.

##### 4.2.1.1 | Introducing the Next Levels of Care.

Nurses introduced the next levels of care by providing detailed information on different levels of healthcare facilities and services. Nurses’ introduction of the next level of care frequently coincided with nurses’ role in facilitating family understanding, where nurses described expectations for patients’ recovery to inform families in making decisions and planning the next levels of care. A nurse communicated the next levels of care, offering details on what to anticipate during the patient’s recovery during the family meeting by stating:

He [patient]’s going to have to progress with his strength and work his way up to another level… Some people transition through one or two levels or sometimes all three… the level we’ll be talking about initially for him is what’s called long-term acute care. What is the average length of stay for those patients? Usually about a month. What is the goal? The goal is to do the weaning, get them off the ventilator. Most patients in long-term acute care have a tracheostomy, they have a feeding tube, they are working on getting off the vent[ilator]… because he’s been on the ventilator for a while … it’s going to be a workout.

(Nurse, #14)

##### 4.2.1.2 | Identifying/Engaging Family Members.

Nurses also identified and engaged family members by establishing relationships with the families and encouraging families to participate when making decisions related to treatment plans, goals and ongoing care. Nurses asked families to bring in any additional family members so that ‘everybody comes in together’ (Nurse, #15) to make decisions. Nurses also emphasised how family members can help patients’ recovery by informing providers about patients’ values, goals and preferences.

##### 4.2.1.3 | Providing Patient/Family Education.

Nurses educated families about their family members’ medical conditions, medications and treatment plans. Family education frequently coincided with nurses facilitating family understanding of the patient’s conditions or prognosis to convey the perspectives of healthcare providers.

*[LTAC]* is sort of like the highest level. That is a doctor sees them every single day, … the nursing care is, it’s more than just two to one … Usually, you’re one of four or five patients… they focus on getting him up out of bed for a couple hours, maybe at this point he’s off the ventilator for a few hours, speech will work with him … building up his strength.

(Nurse, #14)

##### 4.2.1.4 | Managing Medications.

Nurses engaged in medication management, helping families understand the medications for patients, including usage and potential side effects. When coordinating care for a patient and working with allied healthcare providers, nurses evaluated how patients took the medication prior to their hospitalisation to make plans for healthcare referrals. For example, one nurse assessed the patient’s medication to make plans for mental health referrals, asking if ‘she [the patient] self-medicated [her medication] with alcohol for her nerves’ (Nurse, #6).

##### 4.2.1.5 | Planning for Discharge.

To facilitate discharge planning, nurses collaborated with allied healthcare providers to arrange for a patient’s transition to home or other settings following hospital discharge. In the discharge planning process, nurses often invited perspectives of interdisciplinary healthcare providers, including social workers or case managers, to better meet the needs of patients and families. Nurses assessed numerous factors, such as patients’ premorbid conditions and functional status, expected prognoses and social determinants of health, to guide collaborative decisions on optimal care and coordinate discharge plans aligned with the patient’s progress as well as their values, goals and preferences.

There’s different levels of care. It’s all based on insurance, that’s why I always have *[social worker]* … she’s going to guide you based on what’s going to be the next appropriate step based on what the doctors are telling her, based on what physical therapy is telling her. So, we rely also on all of her ancillary services to tell us what they think he can even tolerate. So, I think she’ll give a little more next week once we know, like I said, which path he, he ends up going down.

(Nurse, #5)

##### 4.2.1.6 | Assessing Patient/Family Needs.

When assessing patient/family needs, nurses gathered information to identify the specific needs (e.g., physical, emotional, spiritual needs), preferences and concerns of patients and their families to tailor plans for patient care and discharge accordingly. Nurses evaluated if patients and families had preferences regarding proximity when determining the location of the next level of care. Additionally, one nurse asked if the patient had spiritual needs and informed that:

We have spiritual care providers here at the hospital if you think [patient] … would like a visit from one of them and have someone to pray for her or pray with her?

(Nurse, #16)

##### 4.2.1.7 | Coordinating Care.

Nurses coordinated care by collaborating with multidisciplinary healthcare team members, comprising physicians, nurses, therapists, case managers, social workers and chaplains to develop individualised plans tailored to the needs of patients and families. For example, one nurse ‘arranged for [*spiritual care*]’ when family members had spiritual needs (Nurse, #16) and invited case manager to coordinate care after discharge:

[*Case manager*] is going to join us and she can talk a little more about what the LTACs are, and what the possibility or options are if there is anything close to where you guys live

(Nurse, #14)

##### 4.2.1.8 | Setting Goals.

Nurses supported families in setting goals to demonstrate progress in recovery and achieve better health. Nurses offered their perspectives on patient recovery to help families establish realistic short- or long-term goals for patient recovery, ‘focusing at least initially on physical [*improvements*] and then working on [*the other remaining domains of recovery*]’ (Nurse, #6).

##### 4.2.1.9 | Providing Care Continuity.

To ensure continuity of care, nurses strived to meet the needs of patients and families consistently without interruptions so that the delivery of healthcare services was streamlined and seamless over time and across different levels of care. Nurses collaborated with social workers who could look into services to support patients’ unique characteristics, such as veteran patients with disabilities, when planning for their eventual discharge to home. Additionally, nurses acknowledged it would be a ‘stepping process’ (Nurse, #6) to ensure care continuity as patients and their families moved from ICU to counselling and rehabilitation services after achieving short-term patient goals, which mostly focused on physical recovery.

##### 4.2.1.10 | Offering Provider Guidance.

As a form of provider guidance, nurses offered advice, recommendations or instructions to ensure the appropriate plan of action for families or other providers. Nurses provided honest and personal directives related to what they would do in this situation. When a family member shared that the children of the patient were presenting changes in their behaviours at home and school because of the patient’s hospitalisation, the nurse mentioned that, if she were in this situation, she would inform the school about the current situation related to the patient’s health and behavioural changes of their children (Nurse, #13). Nurses also demonstrated accountability by indicating that they would make sure to keep ‘all [family members and healthcare providers] on the same page’ (Nurse, #10) throughout the patient’s ICU stay, given the subtle and sometimes explicit differences in opinions across all individuals involved in family meetings.

##### 4.2.1.11 | Referring to Resources.

Lastly, nurses provided recommendations or assisted with obtaining referrals to resources within the healthcare system and community resources. For example, when families asked for support and resources, nurses provided guidance to the family with appropriate services and available resources:

Family: Are you aware of any kind of support or help that the VA offers to veterans that are disabled in the kind of shape he’s in?

Nurse: ‘Our social worker would probably be able to answer that more. Are you thinking more for eventually when he would maybe make it back home?’

(Nurse, #9)

While these strategies were identified and described separately, nurses utilised these strategies iteratively, multiple times, often simultaneously and in no particular order. Their usage depended upon patients’ condition and progress, as well as the questions that the families were asking in the family meetings and directives from other disciplines. Further, nurses’ role as transitional care liaisons intersected with their other roles, including information and communication facilitators and family support providers to provide holistic patient- and family-centred care.

#### 4.2.2 | Theme 2: Nurses as Information and Communication Facilitators

Nurses played a crucial role during the ICU family meeting with allied healthcare providers and family members present, including helping families to understand the patient’s situation, progress and treatment plan, encompassed in three subthemes: (1) moderating family meetings, (2) facilitating family understanding, (3) serving as communication intermediaries.

##### 4.2.2.1 | Moderating Family Meetings.

Nurses dedicated significant effort to guiding discussions in family meetings. Nurses began by facilitating introductions of all individuals present, including themselves, healthcare providers and family members. Further, nurses orchestrated the meetings to ensure the meetings stayed on track and all necessary topics were discussed by explaining and enforcing ground rules, setting expectations at the meeting’s outset and keeping the discussions aligned with the agenda. For example, nurses provided a general idea of how the family meeting will be conducted:

He [physician]’s usually gonna ask you guys to at least, one of you [family members], speak up and say what you think you know is going on and then he’ll give you an update and then we can all talk to see if you guys have questions.

(Nurse, #7)

During the family meeting, nurses redirected the discussions back to the agenda if participants asked questions that would warrant a discussion on a new topic or that were on the agenda to be discussed at a later point in the meeting:

Family: ‘And that’s where you’re talking transitional care?’

Physician: ‘Yes. Exactly, yes’.

Nurse: ‘Yes, there’s different levels and I’m going to talk to you a little more about that when he’s done’.

(Nurse, #14)

Toward the end of family meetings, nurses allowed other healthcare providers (i.e., physicians) to exit the conversation after they had shared their updates so they could move on to other work before wrapping up the family meetings. While wrapping up the meeting, nurses prepared the family for future meetings (i.e., setting future topics) and summarised and shared the agreed-upon plans with families as discussed.

##### 4.2.2.2 | Facilitating Family Understanding.

Nurses worked to ensure that family members understood the information shared by healthcare providers by providing additional (often more simplistic) explanations, conveying perspectives of other providers when communicating patient prognosis and recovery expectations, informing families about progress needed for patients to proceed to the next level of care, sharing concerns other providers have and walking them through the decision-making process. The goals of facilitating family understanding were to fulfil families’ needs, including their information and communication needs, and to provide opportunities for shared decision-making. Often, nurses raised questions families had asked them before or during the meeting to ensure their questions were addressed:

Dr. [physician], can you touch base a little bit. [family member] mentioned about somebody told him about her wearing a mask and I think that we should explain that.

(Nurse, #8)

Additionally, nurses provided time and space for families to think about their decisions and process these decisions with other family members who were not present at the meeting, as one nurse stated:

You go home and you talk to … your brother-in-law and your kids and not everybody can be here, but they would all want to be in that discussion.

(Nurse, #11)

To increase families’ understanding, nurses employed a variety of communication skills during the family meeting. For example, nurses utilised active listening, asked follow-up questions, acknowledged information being shared or provided further explanations, clarifications or information.

Nurses in facilitating understanding of the families often co-occurred as part of the family education related to the patient’s physical and psychological conditions and/or premorbid conditions, the introduction of different levels of care and the involvement of family members.

##### 4.2.2.3 | Serving as Communication Intermediaries.

Nurses acted as a communication link between families and other allied healthcare providers to allow for reconciliation of the information being shared between families or providers; this subtheme often co-occurred with facilitating family understanding. More specifically, nurses validated families’ statements by acknowledging the information they shared. Further, nurses also indicated whether they held similar perspectives to other healthcare providers. When nurses agreed with other providers’ perspectives, they broke down the other providers’ perspectives. For instance, a nurse who agreed with a physician’s perspective shared:

Dr. [physician]’s right; it’s sort of a couple steps forward, a step back.

(Nurse, #14)

On the other hand, when the nurse did not agree with other providers’ perspectives or if the information another provider shared was not up to date, the nurse clarified with the most current information they had on the patient’s condition:

Physician: ‘His heart is working okay as far as we know but certainly the lungs and the abdomen are the big, the big worry right now. I would say that he has, is a little bit worse today than he was yesterday … we’d like to see him getting a lot better every day and we’re not seeing that’.

Nurse: ‘He … was already sitting up for two and a half hours today’.

(Nurse, #19)

#### 4.2.3 | Theme 3: Nurses as Family Support Providers

Nurses offered multidimensional support to families and fostered a supportive environment to address concerns and decisions related to a patient’s care. This theme commonly co-occurred with Theme 2: Nurses as information and communication facilitators. The Nurses as Family Support Providers theme had three subthemes: (1) providing emotional support, (2) describing expectations and (3) advocating for patients and families.

##### 4.2.3.1 | Providing Emotional Support.

By expressing compassion, empathy and reassurance, nurses encouraged families to cope with their feelings, navigate hardships and foster a sense of connection. Nurses often related to families, expressing that they hear the family ‘loud and clear’ (Nurse, #14), and acknowledged the emotions experienced by families, sometimes by saying things like ‘it’s a lot to handle. It’s okay to be upset and scared’. (Nurse, #7). Additionally, nurses reassured families that they would always be there to give the family updates on the patient’s conditions and ensure that their concerns were heard and addressed. Nurses also provided positive reassurance to families, validating the families’ valiant efforts to support patients:

I don’t think you can function any better than what you’re doing right now with as much as you’ve all been through.

(Nurse, #13)

##### 4.2.3.2 | Describing Expectations.

Nurses helped families gain a better sense of what is expected of patients’ conditions and recovery. Nurses’ goal in describing these expectations in advance of happenings was to reduce the shock, anxiety or negative emotions that family members could potentially experience during the patient’s treatment and recovery. One nurse mentioned that she ‘wanted to give you [the family] a heads up as to … what that road could look like’. (Nurse, #14).

##### 4.2.3.3 | Advocating for Patients and Families.

Nurses actively supported and represented patients’ and families’ interests and needs during the patient’s ICU stay. Nurses advocated for patients and families by stating they would be consistently physically present, sharing they would ‘be here’ (Nurse, #13). Nurses also told families they would ‘honor what you [family member] say’ (Nurse, #14) to respect their values, goals, and perspectives and meet patient/family needs so that they could receive appropriate and quality care. Nurses advised families on what they perceived they could do to better support their patients and other family members. For example, nurses recommended families also take care of themselves:

Obviously I don’t know her like you do, but she probably wouldn’t want you to risk your own health trying to do it even if you thought you could.

(Nurse, #2)

## 5 | Discussion

The purpose of this study was to explore the nurses’ role in transitional care planning in ICU family meetings for patients with PMV. Nurses engaged and spoke at length beyond introduction in approximately a quarter of family meetings in which transitional care was discussed. Furthermore, among the family meetings where nurses spoke at length, findings showed nurses served as: (1) transitional care liaisons, (2) information and communication facilitators and (3) family support providers.

One of the key findings of this study and contributions to the literature was ICU nurses’ role as transitional care liaisons. Our findings suggest that nurses served as transitional care liaisons trying to initiate discussions around transitional care planning through the use of numerous strategies, such as seeking discharge options, coordinating care and assessing patient/family needs. In doing so, nurses were able to allow families to begin thinking about their values and goals so they could make important transitional care decisions without feeling rushed. Early exposure to transitional care planning may help ICU patients and their families to make sense of the situation and provide reassurance, because they are expected to go through multiple ‘critical junctures’ and different levels of care, including nursing facilities, rehabilitation facilities, long-term acute care or home after discharge from the ICU (Davidson 2010; Page et al. 2019). Although prognostic uncertainty creates a barrier to definitive discussions of transitional care planning, the discussions about transitional care possibilities are imperative to give families an opportunity to contemplate their values, goals and preferences, negotiate realistic and unrealistic expectations and resolve misconceptions about patients’ recovery regarding physical functioning and cognitive level after hospital discharge (Turnbull et al. 2016).

Our finding regarding nurses serving as information and communication facilitators was consistent with previous research that noted the overall role of ICU nurses is to act as liaisons and translators between other healthcare providers and the patient and their family members (Kryworuchko et al. 2013; Pecanac and King 2019; Wubben et al. 2021). Not only addressing logistical procedures, nurses often play a mediation role as they are the ones who interact with families the most on a daily basis, communicating contextual information for treatment decisions based on the patient’s values, goals and preferences, delivering messages to both providers and family members that continue outside of family meetings (Cai et al. 2015; DeKeyser Ganz et al. 2016; Garrouste-Orgeas et al. 2016). Additionally, the findings of this study were in line with previous reports of the communication skills that nurses use to communicate with families in critical care settings (Adams et al. 2017). As nurses play a crucial role in communicating with family members as well as providers throughout the patient’s ICU stay, effective communication strategies can contribute to successful transitional care for patients after ICU discharge, resulting in better satisfaction in care (Mitchell et al. 2018). The present study contributes to the literature by providing additional evidence of nurses utilising a variety of communication skills, such as active listening, questioning, acknowledging and clarifying information being shared, to better engage families of patients with PMV in family meetings with providers, which is fundamental to shared decision-making and patient- and family-centred care.

However, we found that nurses spoke beyond introducing themselves in only 25% of the family meetings in our sample. Existing literature corroborates our finding that nurses’ participation in family meetings was low even though they are likely to spend a large amount of time and communicate frequently with family members (Adams et al. 2017; Pecanac and King 2019). In our sample, nurses who engaged at length were primarily nurse coordinators or nurses with unspecified roles; only three (13%) were confirmed bedside nurses and their contributions were minimal. This distinction is important, as nurse coordinators may have responsibilities and communication roles that differ from bedside nurses, potentially influencing the types of themes identified. Institutional policies and norms, such as routine inclusion of nurse coordinators in family meetings at one site, could also help promote nurse engagement. This study adds to the literature on the need for the representation and engagement of nurses, including different nursing roles, to provide holistic perspectives as part of the interdisciplinary team providing consistent and direct care to patients (Wysham et al. 2017). These findings underscore the need to explore how different nursing roles can contribute meaningfully to transitional care planning and suggest that further research is needed to understand how best to engage bedside nurses in ICU family meetings.

A theme identified in this study is that nurses provide multidimensional support for ICU patients and families, similar to what is documented in the literature (Cuzco et al. 2021; Davidson 2010; McAndrew et al. 2020; Minton et al. 2020). In particular, families of ICU patients experience anxiety, stress, depressive symptoms, and fear, which can persist for years after the patient is discharged from the hospital (Davidson et al. 2012; Herridge and Azoulay 2023). Moreover, emotional support needs and informational needs are some of the most commonly reported unmet needs of family members (Dale et al. 2020). Nurses are adept at providing emotional support through empathic communication, which can alleviate symptoms of anxiety and stress in family members (Dale et al. 2020). Moreover, nurses are recommended as coaches assisting family members in decision-making and providing support to families of critically ill patients along the care continuum (Gainer et al. 2017; You et al. 2024).

To coordinate better care and improved care delivery, research suggests transitional care planning should begin early, such as during the ICU stay (Peters 2017). Yet, there is limited knowledge of the role of nurses in ICU transitional care. ICU patients and their families report limited preparation and guidance for hospital discharge, which perpetuates further complications and unmet needs (Deacon 2012; Donaghy et al. 2018; Page 2016). Moreover, the gap between acute hospital care and community-based care is often exacerbated when there is poor communication and care coordination, namely, a lack of transitional care planning (Donaghy et al. 2018).

### 5.1 | Implications for Research and Clinical Practice

To overcome the limitations of a secondary analysis where the overarching study design of the parent study informed data collection and study findings, future research should focus on gaining a deeper understanding of nurses’ role in ICU transitional care. This research could include facilitators and barriers for nurses discussing transitions of care in ICU family meetings to enhance communication effectiveness and to identify patient and family needs as well as the needs of the nurses caring for and supporting them. Moreover, as transitional care planning discussions are iterative processes that occur throughout the patient’s ICU stay and involve an interdisciplinary approach, future studies would need to consider interdisciplinary collaboration and various contextual factors that can come into play. These inquiries could result in a better understanding of the roles and responsibilities of nurses in delivering supportive communication, as well as improving transitional care planning strategies for nurses to support families in the ICU. Findings can be used to develop nursing-led strategies to improve transitional care planning among patients with PMV.

In order to provide an optimal environment for nurses as part of interdisciplinary collaboration in discussing transitions of care for ICU patients and their families, individual-, unit- and hospital-level training and education for nurses, as well as organisational support, would be essential. In clinical settings, increased representation of nurses and their active engagement in discussions about transitions of care in ICU family meetings are needed to improve overall patient and family experiences. Additionally, providing education and training for nurses is essential to support the role of nurses in family meetings and engaging in interprofessional communications and collaboration for shared decision-making. Beginning the transitional care discussions early in the ICU, to make treatment decisions that reflect patient and family values and preferences, and continuing on as the patients move across the care continuum may ultimately lead to better health outcomes. The development of US transitional care guidelines is sorely needed to inform the role of nurses in ICU transitional care.

### 5.2 | Strengths and Limitations

Strengths of this study include analysing less commonly available data and unstructured family meetings, which allowed us to capture nurses’ engagement in transitional care discussions in ICU family meetings. Moreover, this study provided foundational knowledge by describing the content discussed and how nurses engaged in family meetings.

This study is not without limitations. This study only focused on nurses’ engagement in family meetings, so we are missing a holistic view of the engagement of all interdisciplinary healthcare providers to enhance the understanding of the nurses’ roles in ICU family meetings and interdisciplinary collaboration. Nurse participation may have been influenced by the awareness that meetings were being audio recorded. Some nurses may have been more reserved or, conversely, more intentional in their contributions due to the recording. However, because the meetings were part of a broader clinical trial focused on a decision aid and the structure was unstructured and naturalistic, the recording is unlikely to have significantly altered the meeting dynamics. Moreover, most of the nurses who spoke at length were nurse coordinators, which may have influenced the nature of the roles and tasks identified in our themes. However, because qualitative research prioritises information-rich cases, these findings may still offer meaningful insights into nursing contributions during ICU family meetings. Further research is warranted to explore how these roles may vary by nursing position and institutional context. Lastly, this study was cross-sectional in nature, but transitional care decisions are not likely to be made in a singular family meeting, warranting future longitudinal research to fully describe the nurses’ role in family meetings. Because we did not conduct a comparative analysis across meetings with and without active nurse participation and engagement, we cannot determine whether the identified themes are exclusive to nurses present at meetings or whether other clinicians might have fulfilled similar roles. Future studies are needed to assess the distinct contributions of different team members to transitional care discussions and how families might respond differently.

## 6 | Conclusion

This study explored the role of nurses in transitional care planning in ICU family meetings for families of patients with PMV. The findings highlighted several roles that nurses served in ICU family meetings, including serving as information and communication facilitators, family support providers and transitional care liaisons. Findings from this study suggest opportunities for improvement in nurse engagement in ICU family meetings through approaches not currently being implemented. Future research should delve into nurses’ roles in ICU transitional care further, focusing on facilitators and barriers in their discussions during family meetings to ensure continuity of care. Individual-, unit- and hospital-level training, education and organisational support for nurses are essential to optimise nurses’ role in ICU transitional care planning.

## Supplementary Material

Supplement

Additional supporting information can be found online in the Supporting Information section. Data S1: jan70191-sup-0001-Supinfo.docx.

## Figures and Tables

**TABLE 1 |:** Family meeting characteristics.

Variables	ICU family meetings (*N* = 19)
Length of family meeting, median (IQR), in minutes	47 (35–55) (Range: 23–69)
Number of nurses present per meeting, median (IQR)	1 (1–1) (Range: 1–3)
Number of family members present per meeting, median (IQR)	3 (3–5) (Range: 1–12)
Type of nurse, *n* (%)	Total number of nurses (*N* = 23)
Nurse coordinator	12 (52.2%)
Bedside nurse	3 (13.0%)
Unspecified	8 (34.8%)
Number of family meetings each nurse attended, median (IQR)	1 (1–2) (Range: 1–6)
Instances of nurses’ speech per meeting, median (IQR), times	13 (9–22) (Range: 1–108)
Number of words nurses	561 (250–929)
spoke, median (IQR), times	(Range 50–3339)
Topics discussed related to transitional care planning,^a^ *n* (%)	
Discharge needs	17 (89.5%)
Discharge dispositions	19 (100.0%)
Functional status	16 (84.2%)
Post-discharge quality of life	17 (89.5%)
Site, *n* (%)	
Site 1	1 (5.3%)
Site 2	13 (68.4%)
Site 3	1 (5.3%)
Site 4	4 (21.1%)

a Categories are not mutually exclusive.

**TABLE 2:** | Meeting participant characteristics.

Characteristics	Patient (*N* = 19)	Family member (*N* = 19)	Nurse (*N* = 12)
Age, in years, mean (SD)	55.5 (12.84)	51.1 (12.26)	43.5 (10.81)
Missing	—	1	4
Female gender, *n* (%)	9 (47.4%)	9 (50.0%)	10 (100.0%)
Missing	—	1	2
Race, *n* (%)			
White	10 (52.6%)	10 (55.6%)	8 (100.0%)
Black	8 (42.1%)	7 (38.9%)	0
Other	1 (5.3%)	1 (5.6%)	0
Missing	—	1	4
Ethnicity, *n* (%)			
Non-Hispanic/Latino	19 (100.0%)	18 (100.0%)	9 (100.0%)
Missing	—	1	3
Relation to patient, *n* (%)	—		—
Spouse or partner	—	8 (44.4%)	—
Child	—	4 (22.2%)	—
Parent	—	1 (5.6%)	—
Sibling	—	2 (11.1%)	—
Other family (e.g., niece, ex-wife, son’s girlfriend)	—	3 (16.7%)	—
Missing	—	1	—
ICU type, *n* (%)		—	—
Medicine	15 (78.9%)	—	—
General surgery	1 (5.3%)	—	—
Neurosurgery	2 (10.5%)	—	—
Neurology	1 (5.3%)	—	—
Illness severity score,^a^ mean (SD)	25.2 (5.85)	—	—
Comorbidity score,^b^ mean (SD)	3.9 (2.68)	—	—

Abbreviation: ICU, intensive care unit.

a Illness severity was measured by APACHE II (Acute Physiology and Chronic Health Evaluation II) (Knaus et al. 1985), with higher scores representing greater illness severity.

b Comorbidity was measured by the Charlson Comorbidity Index (Quan et al. 2011), with higher scores representing more comorbidities.

## Data Availability

The data that support the findings of this study are available from the corresponding author upon reasonable request.

## Summary

- What does this paper contribute to the wider global clinical community?
  - Highlights how nursing involvement can improve communication and continuity of care.
